# Incidence of Legionnaires’ Disease among Travelers Visiting Hotels in Germany, 2015–2019

**DOI:** 10.3201/eid3001.231064

**Published:** 2024-01

**Authors:** Udo Buchholz, Bonita Brodhun, Ann-Sophie Lehfeld

**Affiliations:** Robert Koch Institute, Berlin, Germany

**Keywords:** Legionnaires’ disease, travel-associated Legionnaires’ disease, hotels, incidence rate, clusters, bacteria, respiratory infections, Germany

## Abstract

We determined whether the incidence rates of travel-associated Legionnaires’ disease (TALD) in hotels in Germany increased after a previous occurrence and whether control measures required by the European Legionnaires’ Disease Surveillance Network after a cluster (>2 cases within 2 years) restored the rate to baseline. We analyzed TALD surveillance data from Germany during 2015–2019; a total of 307 TALD cases (163 domestic, 144 nondomestic) in hotels were reported. The incidence rate ratio was 5.5 (95% CI 3.6–7.9) for a second case and 25 (95% CI 11–50) for a third case after a cluster had occurred, suggesting that control measures initiated after the occurrence of TALD clusters might be inadequate to restore the incidence rate to baseline. Our findings indicate that substantial LD preventive measures should be explored by hotels or other accommodations after the first TALD case occurs to reduce the risk for future infections.

Legionnaires’ disease (LD) is caused by bacteria of the genus *Legionella*, predominantly by *Legionella pneumophila* serogroup 1. Humans are infected via contaminated aerosols. The list of confirmed infection sources is long and includes drinking water piping systems, evaporative condensers, and whirlpool spas (*1*). Usually, proof of an infection source for individual cases is difficult. However, exposures within 2–10 days (incubation period) before symptom onset are categorized as community-acquired LD cases (≈75% of all cases in Germany), travel-associated LD (TALD) cases (≈20% of all cases in Germany), or hospital/healthcare–acquired LD cases (≈5% of all cases in Germany) (*2*). Whereas large LD outbreaks are rare, TALD clusters occur frequently.

TALD cases are associated with hotels or other commercial accommodations (e.g., campsites or holiday apartments). Those accommodations are often at higher risk because they frequently have complex water systems, might be periodically unoccupied, and sometimes offer additional facilities, such as whirlpool spas, to their guests; all of those factors are associated with an increased risk for LD (*3*,*4*).

In Europe, TALD cases are reported by national public health authorities to the European Legionnaires’ Disease Surveillance Network (ELDSNet, https://www.ecdc.europa.eu/en/about-us/partnerships-and-networks/disease-and-laboratory-networks/eldsnet) that is hosted by the European Centre for Disease Prevention and Control in Stockholm, Sweden. ELDSNet collects TALD case data and informs countries about commercial accommodations that persons with LD had visited. Since the end of 2012, Germany has participated in ELDSNet and reports TALD cases for residents of the country who have been associated with a commercial accommodation in Germany or abroad.

In 2012, the TALD incidence rate in Europe was estimated at 0.3 cases/1 million nights (*5*). When restricting those data to countries that reported the most cases of TALD to ELDSNet (implying that they reported more completely than other countries), results suggested that the incidence rate among nondomestic travelers (travelers from outside of the country where the hotel was located) might be 2-fold higher on average.

According to ELDSNet, a TALD cluster is defined as a commercial accommodation where >2 case-patients with TALD stayed within 2 years and LD developed within 2–10 days after their stay. After a cluster is reported, local health departments responsible for the respective commercial accommodations are required to initiate an investigation that includes creating a risk assessment, taking environmental samples, and introducing control measures, if deemed suitable, such as thermal disinfection, cleaning, or structural improvements (*6*). The health department judges the adequacy of control measures and confirms that they were initiated appropriately (*6*). Within accommodations where a TALD cluster had occurred, the risk for a further case (reoffender) was estimated at ≈12.4/100 accommodation-years (*3*). Another study found that the probability of a second TALD case at the same accommodation site varies according to the country and size of the hotel (*7*).

Examining the incidence rate of TALD in hotels with 0, 1, or 2 previous LD cases by using person-time as the denominator would be useful to investigate whether the incidence rate for TALD among hotels with a first case might be higher than baseline. In addition, preventive measures taken after the occurrence of a cluster might substantially reduce the incidence rate for further TALD cases. Knowing the values of those indicators might enhance the evidence base used for recommendations on managing accommodations that have 1 or 2 LD cases. Thus, the objectives of this study were to estimate the incidence rate of all TALD cases associated with hotels in Germany in general (traveler incidence), estimate the incidence rate of hotels in Germany that had their first LD case after the start of the study period (January 1, 2015), evaluate the incidence rates among accommodations after 1 or 2 LD cases occurred, and determine the incidence rate when 2 cases were associated with the same accommodation within 2 years (cluster).

## Methods

Similar to other countries in Europe, LD is notifiable in Germany. As part of the notification process, the patient's exposure history, particularly travel history, is also reported. We analyzed data on accommodations in Germany that were associated with >1 case-patient with TALD who resided in Germany or abroad during 2015–2019. We selected those dates because TALD case data were still relatively incomplete before 2015, and the COVID-19 pandemic had started and influenced travel behavior of the population after 2019 (*8*). We restricted analyses to case-patients with TALD who had stayed in hotels, guesthouses, or boarding houses (hereinafter hotels) and excluded ships, campsites, and other types of commercial accommodations, such as holiday apartments. If a case-patient had stayed in >1 hotel, each hotel was categorized as having been associated with a TALD case.

We assumed that hotels had a stable bed capacity (i.e., we assumed no structural changes had occurred during 2015−2019). Moreover, we assumed that the occupancy rate was constant and consistently applied to all accommodations. According to 2017 data from Statista (https://www.statista.com), we assumed a conservative general bed occupancy rate of 70% (*9*).

We calculated 4 indicators. The first indicator was the number of all cases of TALD/number of nights spent by all travelers in hotels in Germany during 2015–2019 (traveler incidence). The second indicator was the number of hotels in Germany that had a first TALD case/number of nights spent by all travelers either until a first case occurred or, for hotels without a case, until the end of the 2019 observation period (first-case incidence). The third indicator was the number of hotels in Germany that had a second TALD case/number of nights spent in those hotels after the first case and until the occurrence of a second case or, for hotels without a further case, until the end of 2019 (second-case incidence). The fourth indicator was the number of German hotels that had a third TALD case/number of nights spent in those hotels after the second case until the occurrence of a third case or, for hotels without a further case, until the end of 2019 (third-case incidence). We also calculated the third-case incidence for hotels that previously had 2 cases within 2 years (defined as a cluster or reoffending hotel). 

**Figure 1:**
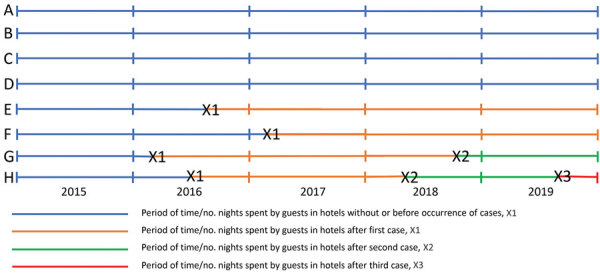
Schematic diagram showing typical timelines used to calculate first-, second-, and third-case incidence rates for Legionnaires’ disease among travelers visiting hotels in Germany, 2015–2019. Data were collected from 29 countries that reported cases of travel-associated Legionnaires’ disease (TALD) to the European Legionnaires’ Disease Surveillance Network (https://www.ecdc.europa.eu/en/about-us/partnerships-and-networks/disease-and-laboratory-networks/eldsnet) after stays in hotels in Germany. Reports were from 26 European Union countries (Cyprus and Slovakia were excluded), Norway, Switzerland, and the United States. A–H indicates different hotels; X1 shows the occurrence of first TALD cases, X2 second TALD cases, and X3 third TALD cases. LD, Legionnaires’ disease.

The 4 indicators were calculated as the number of TALD cases or number of hotels that had a TALD occurrence per million nights spent in hotels in Germany. To calculate indicators, we searched Eurostat (https://ec.europa.eu/eurostat) to identify the number of nights that guests spent in hotels in Germany during 2015–2019; we searched all countries that reported TALD incidents to ELDSNet, which included 26 European Union countries (Cyprus and Slovakia were excluded) plus Norway, Switzerland, and the United States, for a total of 29 countries (designated all-hotel-nights-29 for calculations) (*10*). Data from those countries comprised 94% of all nights that guests stayed in hotels in Germany. For the first indicator (traveler incidence), we divided the total number of TALD cases by the number of all-hotel-nights-29. In addition, we calculated the traveler incidence separately for domestic travelers (residents of Germany) and nondomestic travelers (residents of 28 countries reporting to ELDSNet other than Germany) as well as for travelers from those countries reporting >10 TALD cases that included a stay in a hotel in Germany. For the second indicator (first-case-incidence), we needed to subtract the number of nights among hotels that had a first case from all-hotel-nights-29. To perform this subtraction, we researched the bed capacity of every hotel that was associated with >1 TALD case. In some hotels, we estimated the number of beds on the basis of the number of rooms. Because we knew the date when the cases occurred, we could calculate the number of nights that guests spent in hotels after the first TALD case before the end of 2019. To determine the number of nights in hotels after the occurrence of a first case (spent in hotels in Germany by visitors only from the countries reporting to ELDSNet), we estimated this number as: bed capacity of all hotels with a first case × 70% occupancy rate × number of days after the occurrence of the first case × 94%; we then subtracted that number from all-hotel-nights-29 to obtain the denominator for indicator 2 (Figure 1). The numerator was the number of hotels with a first case during 2015−2019.

To estimate the third (second-case-incidence) and fourth (third-case-incidence) indicators, we used a similar concept (Figure 1). For the third indicator, we calculated the denominator as the number of hotel nights after the occurrence of a first case until the end of 2019 or until the occurrence of the second case. The numerator was the number of hotels with a second TALD case. For the fourth indicator, we calculated the denominator as the number of hotel nights after occurrence of a second case until the end of 2019 or until the occurrence of the third case. The numerator was the number of hotels with a third TALD case.

We also calculated the third-case incidence among hotels that had TALD clusters (reoffending hotels), where measures deemed necessary had been taken to prevent further cases. ELDSNet permits 6 weeks for health departments and hotels to take preventive measures; we added another 2 weeks to account for the *Legionella* incubation period. Thus, we calculated the risk of having another TALD case >8 weeks after the cluster notification.

To examine hotel size as a potential confounding or effect-modifying factor, we stratified the second-case and third-case incidences according to hotel size (<200 beds was small, >200 beds was large) and compared those incidence rates. This calculation was not possible for traveler or first-case incidence rates because we did not know the distribution of nights spent among small and large hotels.

Sampling results from on-site investigations were mainly available for hotels associated with >2 cases. In addition, *Legionella* sp. strains were only sporadically typed (e.g., by using monoclonal antibody subtyping).

## Results

During 2015–2019, a total of 384 TALD cases (case-patients residing in Germany or abroad) associated with any commercial accommodation in Germany were reported. Of those, 307 TALD cases were associated with hotels; 163 were domestic and 144 nondomestic cases (Table 1). Of the 144 nondomestic cases, 62 were reported from the Netherlands, 14 from Denmark, and 13 from France; the remaining cases were reported from 13 other countries. The 307 case-patients named 357 hotels, which partially overlapped, for a total of 309 different hotels. Of the 307 case-patients, 274 (89%) stayed in 1 hotel and 33 (11%) stayed in >2 hotels in Germany. The average number of named hotels per case was 1.2 (range 1−6). The number of TALD cases in hotels in Germany increased from 22 cases in 2015 to 94 cases in 2019. 

**Table 1:** Main characteristics of case-patients and hotels in study of incidence of Legionnaires’ disease among travelers visiting hotels in Germany, 2015–2019*

Characteristics	All TALD cases	Domestic TALD cases	Nondomestic TALD cases	p value
No. case-patients	307	163	144	
Age, y†				
<60	103 (34)	59 (36)	44 (31)	
>60	203 (66)	104 (64)	99 (69)	0.32
Sex†				
M	238 (78)	130 (80)	108 (76)	
F	68 (22)	33 (20)	35 (24)	0.38
No. visited hotels				
1	274 (89)	147 (90)	127 (88)	
>1	33 (11)	16 (10)	17 (12)	0.57
Median (mean; range)	1 (1.16; 1–6)	1 (1.15; 1–4)	1 (1.18; 1–6)	0.58
Hotel size‡				
<50 beds	109 (30)	66 (35)	43 (25)	
51−199 beds	135 (38)	70 (37)	65 (38)	
>200 beds	113 (32)	51 (27)	62 (37)	0.07
Median (range) no. beds	90 (4–1,920)	76 (4–1,402)	125 (9–1,920)	0.008

*Values are no. (%) except as indicated. Data were stratified according to domestic and nondomestic cases of TALD; nondomestic cases were from any of 28 countries reporting to the European Legionnaires’ Disease Surveillance Network (https://www.ecdc.europa.eu/en/about-us/partnerships-and-networks/disease-and-laboratory-networks/eldsnet) other than Germany. TALD, travel-associated Legionnaires' disease. †No information was available for 1 case. ‡Hotel size was categorized according to the number of beds. Multiple visited hotels per case were possible.

Overall, 103 (34%) case-patients were <60 and 203 (66%) were >60 years of age; 238 (78%) were male and 68 (22%) were female. For 1 case, no information was available for age or gender. Age group, gender, or the number of named hotels did not differ between domestic and nondomestic TALD cases. However, compared with domestic case-patients, nondomestic TALD case-patients visited larger hotels (p = 0.008) (Table 1).

### Hotels Associated with TALD Cases

Of the 309 hotels identified, 281 (91%) were associated with 1 TALD case, 16 (5.2%) with 2 cases, 8 (2.6%) with 3 cases, 1 (0.3%) with 4 cases, 2 (0.7%) with 5 cases, and 1 (0.3%) with 6 cases. The median number of beds was 71 in hotels with 1 case, 159 in hotels with 2 cases, and 208 in hotels with >2 cases. All 309 hotels had a first TALD case (i.e., the hotel was associated with a TALD case for the first time after January 1, 2015), 28 hotels had >2 cases, and 12 hotels had >3 cases. The ratio of domestic:nondomestic cases was 52:48 among hotels when the first case occurred, 50:50 among hotels when the second case occurred, and 67:33 among hotels when the third case occurred. Among hotels with >2 cases, the second case occurred at a median of 375 (range 0−1,559) days after the first case occurred.

### TALD Incidence

**Figure 2:**
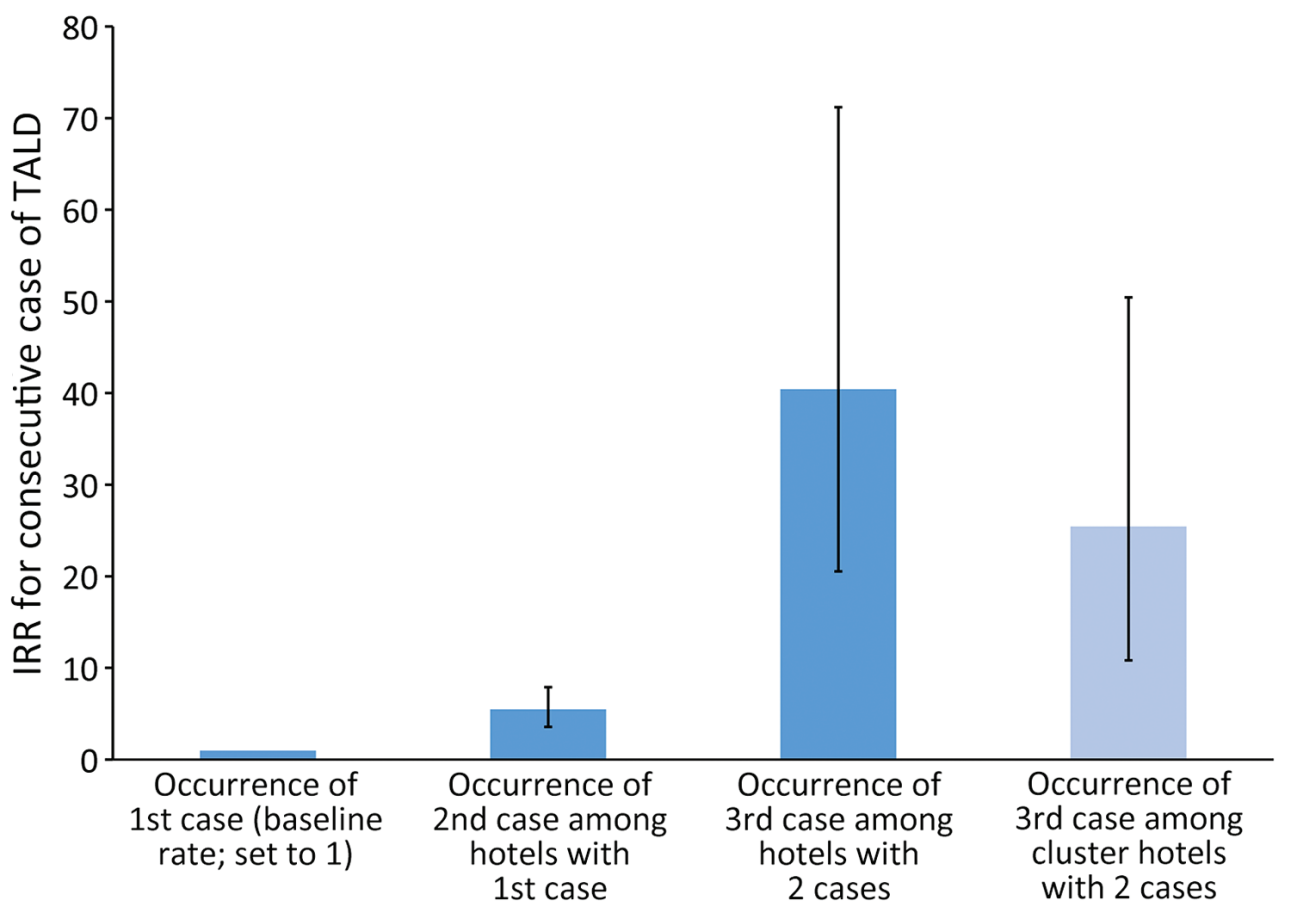
Incidence rate ratios for Legionnaires’ disease cases among travelers visiting hotels in Germany, 2015–2019. IRRs were calculated for hotels that had a second or third TALD case or experienced a cluster of cases. Cluster hotels had 2 cases within 2 years; reoffending hotels were cluster hotels that had 2 cases and another case >8 weeks after the cluster notification. First-case incidence was the referent. Error bars indicate 95% CIs. IRR, incidence rate ratio; TALD, travel-associated Legionnaires’ disease.

During 2015–2019, guests residing in countries reporting to ELDSNet spent 1,351,540,219 nights in hotels in Germany. TALD incidence rate was 0.227/1 million nights (traveler incidence). The first-case incidence (referent) was 0.233/1 million nights, the second-case incidence was 1.3/million nights, and the third-case incidence was 9.4/1 million nights (Table 2). The third-case incidence for hotels that had a cluster according to the ELDSNet definition (reoffending hotels) was 5.9/1 million nights. The incidence rate ratios (IRRs) were 5.5 for second-case incidence and 40 for third-case incidence (Table 2; Figure 2); the difference was statistically significant. 

**Table 2:** Incidence rates for first, second, and third occurrences of Legionnaires’ disease among travelers visiting hotels in Germany, 2015–2019*

Constellation	No.	TALD cases/1 million nights	IRR (95% CI)
First-case incidence rate†			
No. nights in hotels in Germany during 2015−2019	1,351,540,219	NA	NA
No. nights from first TALD case until end of 2019	24,568,957	NA	NA
No. nights in hotels that did not have a TALD case or until occurrence of first case	1,326,971,262	NA	NA
No. hotels that had a first TALD case	309	NA	NA
First-case incidence	NA	0.23	Referent
Second-case incidence rate among hotels that had a first case			
No. nights among hotels that had a first case before the end of 2019 or until the occurrence of a second case	21,905,432	NA	NA
No. hotels that had a second TALD case	28	NA	NA
Second-case incidence	NA	1.3	5.5 (3.6−7.9)
Third-case incidence rate among hotels that had a second case			
No. nights among hotels that had a second case before the end of 2019 or until the occurrence of a third case	1,274,759	NA	NA
No. hotels that had a third TALD case	12	NA	NA
Third-case incidence	NA	9.4	40 (21−71)
Third-case incidence rate among cluster or reoffending hotels			
No. nights among cluster hotels before the end of 2019 or until the occurrence of a third case >8 wk after cluster notification	1,350,676	NA	NA
No. reoffending hotels that had a third TALD case	8	NA	NA
Reoffender incidence	NA	5.9	25 (11−50)

*Data were reported by 29 countries (including Germany) to the European Legionnaires’ Disease Surveillance Network (https://www.ecdc.europa.eu/en/about-us/partnerships-and-networks/disease-and-laboratory-networks/eldsnet); numbers of nights are provided for travelers of those 29 countries. IRR, incidence rate ratio; NA, not applicable; TALD, travel-associated Legionnaires’ disease. †Baseline/referent incidence rate among all hotels.

The traveler incidence rate was 0.15/1 million nights for domestic travelers and 0.58/1 million nights for nondomestic travelers staying at hotels in Germany. The traveler incidence rate was 2.05/1 million nights for persons from the Netherlands, 1.12/1 million nights for travelers from Denmark, and 0.89/1 million nights for travelers from France.

Among smaller hotels (<200 beds) that had a first case, guests stayed 6,135,216 nights and 15 second cases of TALD occurred (incidence rate, 2.4 cases/1 million nights), whereas, among smaller hotels with a second case, guests stayed 215,433 nights and 6 third cases occurred (incidence rate, 28 cases/1 million nights; IRR, 11 [95% CI 4.2–25]). Among larger hotels that had a first case, guests stayed 15,770,216 nights and 13 second cases occurred (incidence rate 0.82 cases/1 million nights), whereas, among larger hotels with a second case, guests stayed 1,059,327 nights and 6 third cases occurred (incidence rate 5.7 cases/1 million nights); the IRR was 6.9 (95% CI 2.5–15). The IRRs for smaller and larger hotels were not significantly different.

In 5 hotels, monoclonal antibody MAb 3/1–positive *Legionella* strains were detected, but it is unknown how many times the antibody typing was performed. Of those 5 hotels, 3 were associated with >1 case.

## Discussion

According to surveillance data from ELDSNet and Germany, our findings show that a substantially increased incidence rate for TALD was associated with hotels in Germany that had only 1 TALD case-patient during 2015–2019. The incidence rate increased further after 2 TALD cases were associated with a hotel. The incidence rates also increased further among hotels that had a TALD cluster after preventive measures had already been performed.

The per-hotel LD risk has been reported to increase with increasing hotel size (*7*); we also showed that the median size increased for hotels that had second or third TALD cases compared with those that had only a first case. Therefore, the increase in IRR (from baseline to hotels that had a second TALD case) observed in this study might be partially explained if larger hotels had an increased incidence rate. Because we did not know the distribution of nights spent among small and large hotels in Germany for traveler incidence and first-case incidence, we could only calculate the ratio for second-case and third-case incidences. Our finding that the increasing IRR applied to both smaller and larger hotels (with no significant difference) suggests that no effect-modifying factors existed and that our overall findings likely apply to both smaller and larger hotels.

Our overall incidence rate of TALD cases in hotels in Germany (0.227/1 million nights) is a composite of the rates among domestic and nondomestic travelers; the incidence rate for domestic travelers (0.15/1 million nights) was substantially lower than that for nondomestic travelers (0.58/1 million nights). One reason for this difference could be that the TALD surveillance systems of other countries in Europe are more sensitive than the system in Germany. The incidence rate for nondomestic travelers during 2015−2019 was lower than that estimated in 2009 for any accommodation (0.79/1 million nights) (*5*). The lower rate during 2015−2019 might be explained, in part, by an increasing number of younger travelers (*11*), and it is also possible that primary prevention of LD in hotels and other accommodations improved between 2009 and 2015.

In this study, we show increased incidence rates for accommodations that experienced first and second TALD cases. A previous study estimated the TALD incidence rate for all cases per total number of hotel nights but did not determine the incidence rates after first or second cases occurred (*5*). Another study calculated the number of cases among accommodations after >2 LD cases occurred but used accommodation-years in the denominator rather than the number of nights spent (*3*). Because the type of hotel can vary substantially from 1 hotel to another, we used the number of nights spent at the respective site as the denominator in our study.

Knowing that the incidence rates of TALD cases increase with each additional case can inform preventive measures to reduce LD infections. The increased TALD incidence rate observed among hotels that had a first case compared with baseline (IRR 5.5) suggests that risk assessments and taking preventive measures might be beneficial after the first occurrence. Furthermore, the even higher IRR among hotels that experienced a cluster (IRR 25) is inconsistent with the requirement of those hotels to investigate the drinking water system and take preventive measures that are deemed adequate by the local health department. If preventive measures are effective, the incidence rate would be expected to return to baseline. However, according to ELDSNet data, many hotels or accommodations have a high propensity to be associated with >1 further TALD cases after a cluster occurs (*3*,*4*,*12*), suggesting that eradicating virulent *Legionella* strains from drinking water installations might be difficult. Assuming that exposure to *Legionella* strains dwelling in piping systems (and conceding that other factors might also play a role) is constant, we postulate that a large proportion of the human population is immune to virulent *Legionella* bacteria. Also, assuming that hotels with 2 associated cases are contaminated with virulent *Legionella* bacteria and are a source for TALD cases and considering that travelers stay 2 days in a hotel on average (*13*), we postulate that 9.4 persons/500,000 travelers (1 million nights divided by 2) are at risk for LD.

The first limitation of our study is that we used a different method to obtain the denominator for the first-case incidence rate than for the second- and third-case incidence rates. Using the same method would require access to a complete list of all hotels and their bed capacity and multiplying those numbers by the assumed 70% occupancy rate, as was done to estimate the denominator for the second- and third-case incidence rates; however, this information was not available for first-case incidence rate calculations. Nevertheless, we believe that the numbers of nights spent at a hotel in Germany provided by the Eurostat website were accurate and already accounted for a 70% occupancy rate. Second, to obtain denominators for the second- and third-case incidence rates, we had to make certain assumptions regarding hotel size or capacities. Third, we assumed that TALD cases were associated with the hotel that the patient visited, although some case-patients might have acquired their infection elsewhere during their travel or at home. Fourth, we assumed that all reported hotels had no LD cases before 2015. Finally, some TALD cases might not have been detected because exposure history could not be determined or because the case-patient resided in a country that did not participate in ELDSNet. Both of those possibilities could have led to underestimation of the traveler incidence as well as first-, second-, and third-case incidence rates.

In conclusion, we have shown that incidence rates of TALD increase significantly after the occurrence of a first case in hotels in Germany and, to a greater extent, after the occurrence of a second case. Local health departments and hotels should explore substantial LD preventive measures after the first TALD occurrence rather than after a cluster to reduce the infection risk for future guests.
